# Supplementary material for: Feasibility, acceptability, and efficacy of a positive emotion regulation intervention to promote resilience for healthcare workers during the COVID-19 pandemic: A randomized controlled trial
Source: PLoS One. 2024 Jun 24;19(6):e0305172. doi: 10.1371/journal.pone.0305172 (PMC11195972; doi:10.1371/journal.pone.0305172)
Supplement: S1 Table — (DOCX) [file pone.0305172.s002.docx]

Supplementary Table S1. Pre-intervention comparisons of demographics, burnout, and well being variables by usage group

|  | No Usage | | Some Usage | | Full Usage | | p |
| --- | --- | --- | --- | --- | --- | --- | --- |
|  | 230 | | 265 | | 52 | |  |
| Age n(%) with data | 230 | 100% | 265 | 100% | 52 | 100% |  |
| mean (sd) | 42.28 | 10.57 | 43.82 | 10.45 | 47.23 | 10.88 | 0.01 |
| Sex n(%) with data | 228 | 99% | 263 | 99% | 52 | 100% |  |
| Male | 34 | 49% | 29 | 41% | 7 | 10% |  |
| Female | 194 | 41% | 234 | 49% | 45 | 10% | 0.44 |
| Race n(%) with data | 221 | 96% | 256 | 97% | 52 | 100% |  |
| White | 191 | 41% | 229 | 49% | 46 | 10% |  |
| Black | 3 | 19% | 12 | 75% | 1 | 6% |  |
| Asian | 24 | 57% | 13 | 31% | 5 | 12% |  |
| Other | 3 | 60% | 2 | 40% | 0 | 0% | 0.07 |
| Occupation n(%) with data | 228 | 99% | 262 | 99% | 52 | 100% |  |
| Administrative | 41 | 36% | 62 | 54% | 12 | 10% |  |
| Physician | 30 | 43% | 31 | 44% | 9 | 13% |  |
| APN, PA | 18 | 47% | 17 | 45% | 3 | 8% |  |
| RN | 72 | 47% | 70 | 46% | 10 | 7% |  |
| Allied Health | 51 | 41% | 62 | 49% | 12 | 10% |  |
| Other | 16 | 38% | 20 | 48% | 6 | 14% | 0.69 |
| Positive Affect n(%) with data | 230 | 100% | 265 | 100% | 52 | 100% |  |
| mean (sd) | 46.45 | 9.55 | 45.35 | 7.89 | 45.57 | 8.64 | 0.37 |
| Meaning and Purpose n(%) with data | 230 | 100% | 265 | 100% | 52 | 100% |  |
| mean (sd) | 51.26 | 11.06 | 51.46 | 9.61 | 51.48 | 10.9 | 0.97 |
| Depression n(%) with data | 230 | 100% | 265 | 100% | 52 | 100% |  |
| mean (sd) | 50.84 | 7.84 | 51.03 | 6.86 | 50.88 | 7 | 0.95 |
| Anxiety n(%) with data | 230 | 100% | 265 | 100% | 52 | 100% |  |
| mean (sd) | 55.58 | 7.68 | 55.48 | 7.42 | 54.51 | 7.51 | 0.65 |
| Social Isolation n(%) with data | 230 | 100% | 265 | 100% | 52 | 100% |  |
| mean (sd) | 46.22 | 9.48 | 45.97 | 9.01 | 45.73 | 8.85 | 0.92 |
| Burnout (Total) n(%) with data | 230 | 100% | 265 | 100% | 32 | 100% |  |
| mean (sd) | 37.76 | 8.16 | 37.44 | 7.14 | 36.5 | 8.96 | 0.56 |
| Sleep n(%) with data | 230 | 100% | 265 | 100% | 52 | 100% |  |
| mean (sd) | 57.3 | 7.69 | 57.47 | 7.44 | 58.63 | 5.99 | 0.5 |
