# Supplementary material for: Feasibility, acceptability, and efficacy of a positive emotion regulation intervention to promote resilience for healthcare workers during the COVID-19 pandemic: A randomized controlled trial
Source: PLoS One. 2024 Jun 24;19(6):e0305172. doi: 10.1371/journal.pone.0305172 (PMC11195972; doi:10.1371/journal.pone.0305172)
Supplement: S2 Table — (DOCX) [file pone.0305172.s003.docx]

| **Supplemental Table 2. Baseline Moderators of PARK effects on Positive Affect** | | | | | | | | | | | | | | | |
| --- | --- | --- | --- | --- | --- | --- | --- | --- | --- | --- | --- | --- | --- | --- | --- |
|  | **Baseline Meaning and Purpose** | | | **Baseline Depression** | | | **Baseline Anxiety** | | | **Baseline Social Isolation** | | | **Baseline Burnout** | | |
|  | **Model Results** | | | **Model Results** | | | **Model Results** | | | **Model Results** | | | **Model Results** | | |
| **Effect** | **Estimate** | **StdErr** | **p** | **Estimate** | **StdErr** | **p** | **Estimate** | **StdErr** | **p** | **Estimate** | **StdErr** | **p** | **Estimate** | **StdErr** | **p** |
| **Intercept** | 45.17 | 0.41 | <.0001 | 46.75 | 0.43 | <.0001 | 49.57 | 0.54 | <.0001 | 44.05 | 0.47 | <.0001 | 38.21 | 0.79 | <.0001 |
| **Intervention** | -0.20 | 0.59 | 0.74 | -0.35 | 0.60 | 0.57 | 0.32 | 0.76 | 0.67 | -0.84 | 0.68 | 0.22 | -1.56 | 1.20 | 0.20 |
| **Time** | 1.48 | 0.45 | 0.00 | 1.27 | 0.46 | 0.01 | 1.19 | 0.56 | 0.03 | 1.27 | 0.48 | 0.01 | 1.43 | 0.83 | 0.09 |
| **BL moderator** | 5.84 | 0.42 | <.0001 | -7.68 | 0.59 | <.0001 | -6.64 | 0.56 | <.0001 | -5.17 | 0.48 | <.0001 | -6.36 | 0.55 | <.0001 |
| **Intervention*Time** | 0.68 | 0.76 | 0.37 | 0.38 | 0.76 | 0.62 | -0.87 | 0.98 | 0.37 | 1.71 | 0.82 | 0.04 | 3.53 | 1.51 | 0.02 |
| **Intervention*moderator** | -0.09 | 0.57 | 0.88 | -0.50 | 0.82 | 0.54 | -1.07 | 0.83 | 0.20 | -0.98 | 0.68 | 0.15 | -0.70 | 0.81 | 0.39 |
| **moderator*Time** | -0.31 | 0.47 | 0.51 | 0.46 | 0.63 | 0.46 | 0.23 | 0.58 | 0.69 | -0.13 | 0.49 | 0.79 | 0.04 | 0.58 | 0.94 |
| **Intervention*mod*time** | -1.60 | 0.73 | 0.03 | 2.87 | 1.01 | 0.00 | 2.75 | 1.06 | 0.01 | 2.76 | 0.80 | 0.00 | 2.35 | 1.02 | 0.02 |
|  | **Change Estimates** | | | **Change Estimates** | | | **Change Estimates** | | | **Change Estimates** | | | **Change Estimates** | | |
|  | **Low** | **Average** | **High** | **Low** | **Average** | **High** | **Low** | **Average** | **High** | **Low** | **Average** | **High** | **Low** | **Average** | **High** |
| **change for intervention** | 2.09 | 1.17 | 0.26 | -0.72 | 0.93 | 2.57 | -0.92 | 0.36 | 1.64 | 0.29 | 1.60 | 2.91 | 1.38 | 2.53 | 3.67 |
| **change for control** | 1.74 | 1.33 | 0.92 | 0.78 | 1.23 | 1.68 | 0.96 | 1.17 | 1.38 | 1.39 | 1.23 | 1.08 | 1.34 | 1.48 | 1.61 |
