# Supplementary material for: Feasibility, acceptability, and efficacy of a positive emotion regulation intervention to promote resilience for healthcare workers during the COVID-19 pandemic: A randomized controlled trial
Source: PLoS One. 2024 Jun 24;19(6):e0305172. doi: 10.1371/journal.pone.0305172 (PMC11195972; doi:10.1371/journal.pone.0305172)
Supplement: S4 Table — (DOCX) [file pone.0305172.s005.docx]

Supplementary Table S4. Reasons for Not Completing Skill Lessons or Practice (N = 127)

|  | Reasons for not  completing Skill Lessons  N (%) | Reasons for not completing skills practice  N (%) |
| --- | --- | --- |
| Not enough time | 67 (52.76) | 64 (50.39) |
| Forgot to do it | 37 (29.13) | 44 (34.65) |
| Lost interest | 38 (29.92) | 27 (21.26) |
| Daily practice too demanding | N/A | 30 (23.62) |
| Practiced on my own outside of platform | N/A | 18 (14.17) |
| Did not need to practice daily | N/A | 11 (8.66) |
| Email reminders too demanding | 13 (10.24) | 9 (7.09) |
| Completed all | 15 (11.81) | 0 |
| Lack of privacy | 6 (4.72) | 9 (7.09) |
| Got what I needed out of PARK then stopped | 5 (3.94) | N/A |
| Internet connection problems | 1 (0.79) | 1 (0.79) |
| Other | 1. 15.76) | 16 (12.60) |

*Participants asked to select all that applied;
