# Supplementary material for: Feasibility, acceptability, and efficacy of a positive emotion regulation intervention to promote resilience for healthcare workers during the COVID-19 pandemic: A randomized controlled trial
Source: PLoS One. 2024 Jun 24;19(6):e0305172. doi: 10.1371/journal.pone.0305172 (PMC11195972; doi:10.1371/journal.pone.0305172)
Supplement: S1 Fig — (DOCX) [file pone.0305172.s006.docx]

Figure S1. Positive affect Pre- and Post-PARK by usage group
