# Supplementary material for: Feasibility, acceptability, and efficacy of a positive emotion regulation intervention to promote resilience for healthcare workers during the COVID-19 pandemic: A randomized controlled trial
Source: PLoS One. 2024 Jun 24;19(6):e0305172. doi: 10.1371/journal.pone.0305172 (PMC11195972; doi:10.1371/journal.pone.0305172)
Supplement: S1 File — (PDF) [file pone.0305172.s007.pdf]

PROTOCOL TITLE: Northwestern Medicine Healthcare Worker SARS-CoV-2 Serology Study

PRINCIPAL INVESTIGATORS:
Charlesnika Evans, PhD, MPH
Professor of Preventive Medicine
Northwestern University Feinberg School of Medicine
633 N. St Clair, 20th Floor
Chicago Illinois 60611
charlesnika-evans@northwestern.edu

John T. Wilkins, MD, MS
Associate Professor in Preventive Medicine and Medicine-Cardiology
Northwestern University Feinberg School of Medicine
680 N. Lake Shore Drive, Suite 1400
Chicago, IL 60611
j-wilkins@northwestern.edu

VERSION NUMBER: 5.0
VERSION DATE: 04 January 2022
PROGRAM SUMMARY:

Appendix B:

Improving the psychological well-being and burnout due to the COVID-19 pandemic in the Northwestern Medicine (NM) Healthcare Worker SARS-CoV-2 cohort

1. OBJECTIVES

The COVID-19 pandemic has had a catastrophic impact on the world, resulting in the death of millions of people. It has placed an especially immense burden on healthcare workers (HCWs) who report damaged psychological well-being.[1] Recent evidence suggests the psychological impact is worsening as the pandemic persists.[2] The objective of this project is to assess and intervene to improve the psychological well-being among HCWs who have been active throughout the ongoing pandemic response. We will evaluate the acceptability, feasibility and early effectiveness of an online psychosocial wellness intervention designed to reduce burnout and promote psychological wellness among HCWs and describe workplace and personal characteristics associated with stress and burnout.

2. Background

Healthcare systems around the world have faced tremendous stress because of the COVID-19 pandemic. Healthcare workers (HCWs) (ie. physicians, nurses, and support staff), who serve as the foundation of the healthcare system, report high levels of psychological stress and burnout, which will likely worsen as the pandemic continues. The consequences of stress and burnout can reduce quality of life for providers and lead to adverse health behaviors (poor dietary choices, reduced physical activity, increased alcohol intake, increases in weight etc.) among HCWs. In addition, burnout can have dire consequences on healthcare delivery effectiveness including poor quality of care and significant cost implications due to medical errors[1] and HCW absenteeism and turnover.[2] In fact, annual estimates of burn-out related turnover range from $7,600 per physician[3] to >$16,000 per nurse.[4] However, programs focused on reducing burnout in HCWs have the potential to reduce costs to the healthcare system by $5,000 per HCW per year.[4] Maintaining and recovering psychological and behavioral well-being is essential to ensuring we have a workforce that is resilient to acute and ongoing stressors such as the COVID-19 pandemic, ensuring that they are capable of providing the highest level of quality and compassionate care to patients. In this project, we will strengthen the resiliency of the Northwestern Medicine (NM) healthcare system by implementing an online psychological well-being intervention (PARK). We will assess HCW willingness to engage in PARK, which has been shown in other populations experiencing stress (e.g. dementia caregivers, general public coping with COVID-19) to be effective. We will also assess if the PARK is effective in reducing stress and associated-burnout, absenteeism, and intentions to leave the workforce in a subset of 750 persons who have been participating in a study of HCWs at NM since Spring 2020. In the entire cohort, we will measure the psychological well-being, levels of burnout, health behaviors, absenteeism, and plans to leave the workforce at three time periods: the start, middle, and end of the study period and assess whether they differ by HCW

characteristics including gender, race, and role in health care. Results from this study will provide much-needed information: **1)** about the current state of psychological well-being and burnout among NM HCWs, now over 1 ½ years into the pandemic; **2)** on the role of an online wellness intervention to improve well-being during a protracted pandemic; and **3)** about the contribution of PARK to reduce burnout, HCW absenteeism and turnover, and potential impacts on costs. **PARK has the potential to have a significant impact on not only NM HCWs but also to be generalizable to other healthcare organizations for addressing burnout and to contribute to lessons learned on how to support HCWs responding to future pandemics; ensuring resiliency in the healthcare delivery system.** In addition, we will work with our already engaged stakeholder committee to ensure results can provide actionable policy and fiscal insights. Future opportunities will include collaboration with other healthcare systems to expand roll-out of the successful PARK intervention.

[1]Hewitt, D. B., Ellis, R. J., Chung, J. W., Cheung, E. O., Moskowitz, J. T., Huang, R., ... & Bilimoria, K. Y. (2021). Association of surgical resident wellness with medical errors and patient outcomes. *Annals of Surgery*, *274*(2), 396-402.
[2]Panagioti, M., Geraghty, K., Johnson, J., Zhou, A., Panagopoulou, E., Chew-Graham, C., ... & Esmail, A. (2018). Association between physician burnout and patient safety, professionalism, and patient satisfaction: a systematic review and meta-analysis. *JAMA Internal Medicine*, *178*(10), 1317-1331.
[3]Han, S., Shanafelt, T. D., Sinsky, C. A., Awad, K. M., Dyrbye, L. N., Fiscus, L. C., ... & Goh, J. (2019). Estimating the attributable cost of physician burnout in the United States. *Annals of Internal Medicine*, *170*(11), 784-790.
[4]Muir, K. J., Wanchek, T. N., Lobo, J. M., & Keim-Malpass, J. (2021). Evaluating the Costs of Nurse Burnout-Attributed Turnover: A Markov Modeling Approach. *Journal of Patient Safety*.

We propose to study the levels of stress and burnout and associations with health behaviors and health-related absenteeism in an existing longitudinal cohort of 3,569 NM HCWs comprised of frontline providers and support and administrative staff. We will also test the acceptability, feasibility, uptake and early effectiveness of an adapted on-line evidence-based intervention (PARK) to improve psychological wellbeing and levels of job burnout among this group. PARK was **adapted by Dr. Moskowitz from a previously validated wellness intervention** found to improve depression, positive emotion, and other aspects of psychological well-being across a range of populations, but not HCWs. Results from this study will: 1) document psychological well-being, health and cardio-metabolic behaviors, and absenteeism in the entire cohort; 2) assess the feasibility of an intervention, previously shown to be efficacious in other populations; and 3) explore the effectiveness of the intervention by comparing psychological well-being and burnout in those assigned to the intervention compared to those in a waitlist control condition. **We expect that PARK will improve overall stress, burnout, and well-being in NM HCWs**.

3. SPECIFIC AIMS AND STUDY ENDPOINTS

Aim 1: Assess the psychological well-being (depression, anxiety, positive affect, meaning and purpose) and burnout in an existing cohort of 3,569 HCWs working at Northwestern Medicine (NM) during the COVID-19 pandemic using self-report questionnaires.

Hypothesis 1: Higher burnout scores and evidence of poor psychological well-being (high depression, and anxiety and low positive affect and meaning and purpose) will be observed in patient-facing HCWs than in administrators.

Aim 2: Explore the associations of psychological well-being and burnout scores with health and cardio-metabolic behaviors (i.e., sleep, physical activity) and health-related absenteeism.

Hypothesis 2: Higher levels of emotional well-being will be associated with healthy behaviors.

Aim 3: Evaluate the acceptability, feasibility, and effectiveness of the evidence-based **P**ositive **A**ffect **R**egulation s**K**ills (PARK) intervention to improve psychological well-being and reduce burnout among HCWs in a randomized control trial (RCT) with a wait list control.

Hypothesis 3.1: Participants will find PARK acceptable and feasible.
Hypothesis 3.2: Compared to control participants, those who have access to the PARK intervention will show improvements in psychological well-being and burnout.

4. PROGRAM INTERVENTION(S)/INVESTIGATIONAL AGENT(S)
PARK intervention program.
Intervention: 6-Week Self-Guided Program: PARK Positive Emotion Skills: The skills will be delivered over approximately 6 weeks, and individuals can participate from any device and location with internet access. A week will consist of 1-2 days of didactic material and 5-6 days of real-life skills practice and reporting. Table 2 shows the maximum amount of time engaged in the PARK program for any participant is 5 hours over the 6-week period, plus completion of the REDCap surveys assessments (burnout, well-being, health behaviors). The intervention will focus on developing the following skills that will be supplemented by home practice: (1) positive events, capitalizing, gratitude; (2) mindfulness; (3) positive reappraisal; (4) personal strength and achievable goals; (5) and self-compassion.[26] Participants cannot skip ahead and can only progress to the next lesson if they have completed the current one, but they can return to old lessons or exercises if they wish to. In addition, it is important to note that this is a multi-component intervention and if participants find that the first few skills they learn are helpful, they may choose to use those rather than continuing on in the program to learn all the skills.

5. PROCEDURES INVOLVED
Ancillary Study Design: A cross-sectional survey will be conducted with the NM HCW SARS-CoV-2 Cohort to evaluate self-report burnout, psychological well-being, and

cardio-metabolic behaviors. From this cohort, a RCT will be conducted to evaluate the feasibility and acceptability and early effectiveness of the PARK program.

Participants and Recruitment
After baseline measures of burnout, well-being, and health behaviors are obtained, up to 750 participants will be recruited for participation in the PARK intervention from the NM HCW SARS-CoV-2 Cohort via direct email. Based on previous data in a general population sample from well-being interventions implemented by Dr. Moskowitz, approximately 60% of those eligible consented and enrolled in the intervention.

Risks and Benefits for Participants
- The primary risk of participation is mistakenly treating this study as a replacement for mental health care; it is NOT. We are delivering this intervention to complement any potential mental health treatment you may be receiving. This study is not a replacement for seeking medical attention or psychotherapy. Our goal is to provide you with tools to help cope with stress, but we do not promise any improvements on psychological well-being. If you believe you should be consulting a mental health professional, please do so whether or not you proceed with the study. If you provide us with information that shows that you might need extra resources, beyond the PARK program, for coping with stress or other concerns, we will email you a list of sources that you can use to find extra support, if you choose to use it. If you want to find mental health treatment, you can ask your doctor for a referral, or you can contact the national Treatment Referral Helpline at 800-662-4357.
- Inconvenience: Being in the study may sometimes be inconvenient.
- Discomfort. Some of the questions you are asked might make you uncomfortable, as they sometimes center around difficult emotions and emotional situations. You may feel sad, embarrassed, angry, or uneasy. You are free to decline to answer any questions or to stop taking part in any discussions at any time.

6. DATA AND SPECIMEN COLLECTION AND BANKING

Survey data from participants will be collected electronically via REDCap. Baseline survey data will be collected through a REDCap-link sent via email. Self-administered follow-up questionnaires will be emailed monthly via REDCap for the duration of the study.

7. SHARING RESULTS WITH PARTICIPANTS

The results of the study will be shared in aggregate through publications and presentations.

8. STUDY TIMELINE

The table below outlines the schedule of events for the ancillary study.

9. INCLUSION AND EXCLUSION CRITERIA

Participants will be eligible if they are a participant in the NM HCW Serology Study and they provide electronic informed consent to participate.

10. VULNERABLE POPULATIONS

Pregnant women and all employees of Northwestern Medicine are be eligible for this ancillary study.

11. PARTICIPANT POPULATION

Accrual Number	Category/Group	Number to Address the Research Question
Local	Adults	Minimum 750 participants

12. RECRUITMENT METHODS

We will recruit participants from NM HCW SARS-CoV-2 Cohort, which includes 6,510 HCWs from across the NM system (See Overview and Preliminary data above). We anticipate enrolling approximately half of the cohort (n=3,250). Using our established email communications with study participants we will email an invitation to participate in this ancillary study. The email invitation will contain a written description about the study and responses to frequently asked questions. The page will include study contact information and a hyperlink to read, review, and sign a REDCap-based electronic informed consent document. Once the consent is reviewed and signed employees can link to the baseline survey.

13. COMPENSATION FOR PARTICIPATION IN RESEARCH ACTIVITIES

Participants will not be compensated for participation in this study.

14. WITHDRAWAL OF PARTICIPANTS

If a participant decides to withdraw from the ancillary study, then data that have been already collected will not be removed from the program database unless explicitly requested by the participant.

15. POTENTIAL BENEFITS TO PARTICIPANTS

We cannot promise any benefits to participants in this research. However, possible benefits may include learning new ways to deal with the daily stresses. Participants may enjoy learning about and applying the PARK skills to their life and completing the home practice exercises, which are like online journals to log their progress in the course.

16. DATA MANAGEMENT AND CONFIDENTIALITY

Data Management

Electronic data will be stored in the eCRFs maintained using the Northwestern University REDCap platform. Thus, only those with appropriate credentials and password will have ability to enter and view program data. Export rights will be restricted to the study team only, and de-identified data will only be exported when necessary for

reporting and quality control purposes to a restricted ‘fsmresfiles’ location at Northwestern.

Further, we will perform centralized monitoring through Data and Status Quality Reports (DSQRs) and REDCap reporting features. The DSQRs will use the REDCap application programming interface (API) functionality to export the program data, and then restructure and summarize the data using statistical software, such as R or SAS. The output for these DSQRs will be housed on Northwestern University’s secure servers with restricted access to study team members only. The reports will be reviewed and discussed weekly, on average, but once the systems are in place and code generated, the reports may be updated in real-time.

Statistical Design, Analysis by Aim and Sample Size/Power Calculations

Aims 1-2 Approach; Study Design and Participants: A cross-sectional survey will be conducted with the NM HCW SARS-CoV-2 Cohort to evaluate self-report burnout, psychological well-being, and cardio-metabolic behaviors. These data will be used to address Aims 1-2 and as a baseline assessment for the intervention in Aim 3. All currently enrolled participants in the NM HCW SARS-CoV-2 Cohort will be sent the baseline survey via REDCap. Based on our previous response rates, we expect at least 2,498 (70%) to complete the survey.

Measures: Participants will complete a ~15-minute survey with questions on psychological well-being, burnout, health and cardio-metabolic behaviors, and absenteeism. These data will serve as a baseline assessment on the current mental and physical health of HCWs. The cohort will also receive the same assessments at the last follow-up assessment given to the trial participants (Aim 3). Burnout will be measured using the Oldenburg Burnout Inventory (OLBI) which is a validated 16–item inventory which measures affective, physical, and cognitive aspects of burnout across positive and negative framed items of two core dimensions: exhaustion and disengagement (from work). The PROMIS/NIH Toolbox measures of psychosocial well-being will be used to measure psychological well-being using a Computer Adaptive Test (CAT) on anxiety, depression, social isolation, positive affect, and meaning and purpose. Administration of these measures has been shown to be feasible and acceptable to participants facing significant life stress. Health behaviors and health-related absenteeism are directly hypothesized to be influenced by psychological distress and associated with cardiometabolic health including dietary practices, alcohol and other substance use, sleep quality and physical activity/sedentary behavior.[31] We will ascertain health behavior using validated instruments selected from the NIH PhenX toolbox (https://www.phenxtoolkit.org/), which is designed for optimal harmonization across studies. Physical activity will be measured via questions from the Phenxtoolkit on physical activity (https://www.phenxtoolkit.org/protocols/view/150901). Sleep measures will be assessed via a sleep-specific CAT (https://www.healthmeasures.net/index.php?option=com_instruments&view=measure&id=867&Itemid=992)

Alcohol measures will be measured through a 30-day quantity and frequency measure from Phenxtoolkit. (https://www.phenxtoolkit.org/protocols/view/30301?origin=domain

). Nutrition and dietary measures will be measured via the Rapid Eating Assessment for Participants – Short Version (REAPS) questionnaire. The intention to leave work and health-related absenteeism will be measured using questions from a Labor Force Survey from the CDC.

Statistical Analysis: The OLBI is a series of 16 statements in which respondents can agree or disagree – with indication of the degree of agreement or disagreement. There are two subscales amongst the 16 items: i) Disengagement items; and ii) Exhaustion items. A total OLBI score is calculated by summing the two sub-totals. Higher scores indicate a greater level of burnout. PROMIS well-being items are scored on a 5-point scale with options ranging from "not at all" to "very much". Higher scores suggest more positive affect. The NIH tool box standardized scoring methods will be used and automatically scored in the REDCap system using previous methodologies by Dr. Moskowitz. Physical activity is assessed in MET (metabolic equivalent) /minutes week, a continuous variable, sleep quality will be determined using a continuous score with higher values reflecting more sleep disturbances, alcohol will be calculated as drinks per week and dietary composition and behaviors will be assessed across a number of domains including frequency of consuming fruits, vegetables, meats, cereals and grains as well as number of meals/week consumed at home vs take out or in restaurants. Baseline responses to burnout, well-being, and behavior questions will be described using univariate statistics (proportions, continuous). Sub-analyses will explore factors associated with higher or lower scores based on occupation, patient-facing position, COVID exposure (work and home), age, gender, and race/ethnicity using bivariate and

Figure 1. Positive Pathways to Health theoretical model with hypothesized pathways linking PARK to psychological and physical health[36]

Aim 3 approach; Conceptual Framework: The PARK program builds resilience to stress through increased experiences of positive emotion. Based on the Broaden and Build theory and revised Stress and Coping theory, Dr. Moskowitz and her team developed the Positive Pathways to Health theoretical model (Figure 1). This model posits that increased positive emotion is the primary mechanism through

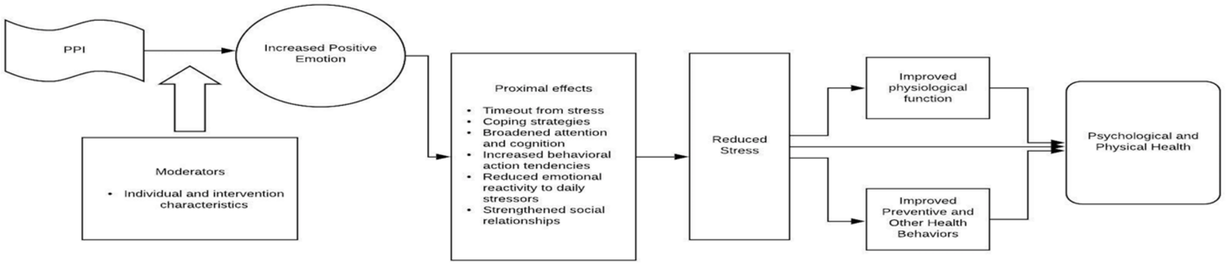

which programs like PARK improve physical and psychological health. Specifically, for HCWs, our theoretical model hypothesizes that engaging in the positive activities in PARK increases the frequency of positive emotion which, in turn, has a range of proximal effects such as providing a timeout from stress,[37] prompting more adaptive coping strategies,[35] reduced emotional reactivity to daily stress, and strengthened social relationships, which all lead to reduced stress, less depression and anxiety, and reduced likelihood of burnout, and subsequently healthy behaviors.
Study Design: A RCT will be conducted to evaluate the feasibility, acceptability, and early effectiveness of the PARK program. The goal of the PARK program is to reduce

feelings of anxiety, depression, and social isolation, as well as increase well-being and a sense of meaning and purpose through the practice of positive emotion skills via a self-guided online platform.

Intervention: 6-Week Self-Guided Program: PARK Positive Emotion Skills: The skills will be delivered over approximately 6 weeks, and individuals can participate from any device and location with internet access. A week will consist of 1-2 days of didactic material and 5-6 days of real-life skills practice and reporting. Table 2 shows the maximum amount of time engaged in the PARK program for any participant is 5 hours over the 6-week period, plus completion of the REDCap surveys assessments (burnout, well-being, health behaviors). The intervention will focus on developing the following skills that will be supplemented by home practice: (1) positive events, capitalizing, gratitude; (2) mindfulness; (3) positive reappraisal; (4) personal strength and achievable goals; (5) and self-compassion.[26] Participants cannot skip ahead and can only progress to the next lesson if they have completed the current one, but they can return to old lessons or exercises if they wish to. In addition, it is important to note that this is a multi-component intervention and if participants find that the first few skills they learn are helpful, they may choose to use those rather than continuing on in the program to learn all the skills.

**Table 2.**	REDCap Survey Assessments	6-week PARK intervention	Total time for PARK
Participant time expected	3 time points=15 minutes each time point	5 minutes/day home practice (3.5 hours total)<br>10-15 minutes/ week of didactic content (1.5 hours total)	**5 hours**

Thus, even though participants might not complete the entire program, they will still experience reductions in stress and burnout, and we plan analyses to explore the effectiveness of partial versus full completion. PARK is delivered through the BrightOutcome online platform. The total time involved from the beginning to the end of the intervention portion of the study is approximately 6 months (includes 6-week PARK course, baseline and follow-up assessments. Wait list controls will be assessed at similar time intervals and will be offered PARK at end of follow-up.

Analysis: We will measure targeted implementation outcomes and explore effectiveness (using similar scoring as described for Aim 1 within and across participants (intervention vs wait list control) and maintenance guided by the RE-AIM framework (Table 3).[38] Change in effectiveness measures within participants will account for repeat measures and differences between intervention and controls will be evaluated using appropriate bivariate and multivariable models. Impact on wait list controls will also be measured after availability. In addition, we will plan sub- analyses based on age, gender, race/ethnicity and job type as well as other potential factors which may influence uptake, completion and effect of PARK.

**Table 3. Outcomes measures using RE-Aim Framework**	
**REACH**	% of eligible HCWs who sign-up; Sub analyses (i.e. by age, gender, race/ethnicity, job)

**Effectiveness**	Change in wellbeing and burnout measures, Difference in measures by intervention and control<br>Change in plans to leave the workforce in the next 6 month
**Adoption**	% of HCWs who sign-up who start PARK
**Implementation**	Feasibility, Acceptability, Fidelity: Ability of HCWs to engage in PARK lessons; % HCWs who complete PARK
**Maintenance**	Persistence of any observed changes in burn-out and well-being 6 months after completing PARK

Sample Size/Power

After baseline measures of burnout, well-being, and health behaviors are obtained, up to 1000 participants will be recruited for participation in the PARK intervention from the NM HCW SARS-CoV-2 Cohort via direct email. Interested individuals will be provided with the URL to complete eligibility screening via REDCap. All HCWs will be eligible to participate unless they have already been identified as a participant in a previous PARK intervention trial (confirmed by Dr. Moskowitz). Once they agree to enroll, they will be randomized to participate in the intervention (PARK) or the ‘wait list control’ arm (No PARK, but will be offered at the end of follow-up). Simple within-group comparison tests would yield 90% power to detect an effect size of d=.20 with a total sample size of 500. Effect sizes in previous tests of the intervention exceeded d = .30. After accounting for attrition and multivariable analyses to assess the effect of PARK between and across intervention and control, a minimum sample of 500 participants will be included. We have opted for a conservative sample size of 750 HCWs from 3,569 enrolled, to be randomized to the intervention or wait list control. As with all the populations we study, those who are interested in reducing their stress will enroll in the program and if it is helping them, they will continue. Interested individuals will be provided with the URL to complete eligibility screening via REDCap. All HCWs will be eligible to participate unless they have already been identified as a participant in a previous PARK trial (confirmed by Dr. Moskowitz). Once they agree to enroll, they will be randomized to participate in the intervention (PARK) or the ‘wait list control’ arm (No PARK, but offered at the end of follow-up).

Limitations

This will be a convenience sample of HCWs who agree to participate and may differ from non-participants.

17. CONSENT PROCESS

The objective of the ancillary study will be described in the initial email to potential participants who are health care workers at Northwestern Medicine that includes a REDCap-based electronic consent form using a digital signature and a video introducing the intervention. Each participant will be informed that participation is voluntary and that they are free to withdraw, without justification, from the study at any time without consequences and without affecting professional responsibilities. We will

ask for consent to: 1) administer our baseline and follow-up surveys, and 2) contact participants about other COVID-related research.

18. PROTECTED HEALTH INFORMATION (PHI AND HIPAA)

The study will collect self-reported protected health information as outlined in the case report form, including related to medical history as well as SARS serological status based on serum specimens. Study participants will also have the option to provide authorization or release of information of protected personal health information but will not be required to do so. This participation will occur through linkage of personal health information to the Northwestern Electronic Data Warehouse (EDW), including:

- Names
- Geographic Subdivisions: All geographic subdivisions smaller than a state, including street address, city, county, precinct, ZIP code and their equivalent geographical codes, except for the initial three digits of a ZIP code if, according to the current publicly available data from the Bureau of the Census: (a) The geographic unit formed by combining all ZIP codes with the same three initial digits contains more than 20,000 people. (b) The initial three digits of a ZIP code for all geographic units containing 20,000 or fewer people are changes to 000.
- Dates and Age: All elements of dates for dates directly related to an individual, including birth date, admission date, discharge date, date of death; and all ages of 89 and all elements of dates (including year) indicative of such age, except that such ages and elements may be aggregated into a single category of age 90 or older.
- Telephone numbers
- Medical Record Numbers
- Medical history, treatment, and other medical record information
- Any other Unique Identifying Number, Characteristic, or Code, unless otherwise permitted by the Privacy Rule for re-identification)
